# Perceived Stress and Internet Addiction Among Chinese College Students: Mediating Effect of Procrastination and Moderating Effect of Flow

**DOI:** 10.3389/fpsyg.2021.632461

**Published:** 2021-06-28

**Authors:** Zhun Gong, Liyun Wang, Haijiao Wang

**Affiliations:** ^1^Department of Psychological Health Education, Normal College, Qingdao University, Qingdao, China; ^2^Qingdao Psychological and Mental Health Research Institute, Qingdao University, Qingdao, China

**Keywords:** internet addiction, perceived stress, flow, procrastination, college students

## Abstract

Perceived stress, Internet addiction and procrastination are common issues among college students. Based on the Compensatory Internet Use (CIU) model and emotion regulation theory, this study aims to explore two possible mechanisms for the connection between perceived stress and Internet addiction: mediating effect of procrastination and moderating effect of flow experience on the Internet. Cross-sectional design and questionnaire survey were used in this study. Data were collected from 446 college students who voluntarily completed self-reporting of perceived stress, internet addiction, procrastination and flow. Potential relationship structure and moderation model between variables was calculated during the process. The results revealed that there were significant associations among perceived stress, Internet addiction, procrastination and flow. The results also showed that procrastination plays a mediating role between perceptual stress and Internet addiction, flow plays a moderating role between them. The results emphasized the importance of the intention behind college students’ overuse of the Internet. These results also provided a perspective of finding the possible causes of Internet addiction in college students, that is, individuals use the Internet to avoid stress and procrastinate, and the mobile experience on the Internet also affects the process.

## Introduction

In today’s world, the Internet closely relates to our life. By March 2020, the world’s Internet penetration rate has reached 58.7%. Approximately 1167% higher than the rate over the past 20 years (Internet World Stats, 2020). Especially for college students, the Internet is an indispensable part of study and life. On the one hand, college students use the network to obtain information and understand the society effectively and extensively; on the other hand, overuse of the network has become a problem that cannot be ignored. The problematic use of the Internet and Facebook is related to lower positive orientation, serious and responsible, emotional stability and openness to experience (Błachnio and Przepiorka, 2016). Severe Internet addiction is also related to depression, low self-esteem and problems with semantic and speech fluency (Nie et al., 2017). In the past two decades, the popularity of Internet devices has aroused people’s interest in researching on the possible consequences of problems caused by Internet-related behavior. One of the problems that studied most is Internet addiction (Young and Rogers, 1998).

Internet addiction is a new form that relies on devices connected to the Internet. This addiction has many negative effects (Khazaei et al., 2017). It is characterized by excessive or poorly controlled obsessions, cravings, or behaviors related to Internet use that lead to conflicts in real life (Ndasauka et al., 2019). Netizens who are habitually obsessed with cyberspace and time, their behavior and psychological state have reached the level of obsession, and it is difficult to extricate themselves. Today’s society has many misunderstandings about the concept of Internet addiction, the intervention and treatment of this addiction, and the medical definition of “Internet addiction” has not yet been fully understood. Most scholars believe that Internet addiction should be referred to as excessive use, abuse or pathological use of the Internet, as a broader category. Because of this, more and more scholars and clinicians are investing in the research of Internet addiction (Laconi et al., 2014).

Influenced by society, family and school, contemporary college students are faced with various pressures in many aspects, which are often overlooked and lead to a certain degree of negative emotional state. There is a significant positive correlation between stressful life events and Internet addiction (Yadav et al., 2013; Zhao et al., 2017). College students’ psychological development is not mature enough. We can make reasonable guesses, which to a great extent may lead to poor emotional control and stress resistance. At the same time, as a large group exposed to the Internet, college students are more likely to rely on the Internet, and relieve stress through the Internet, resulting in Internet addiction. When such maladaptive cognitions have been established, they can further adversely affect the extent of the students’ degree of internet addiction (Han et al., 2017). The current literature on the relationship between stress and Internet addiction is mainly divided into two categories: one is that Internet addiction is related to stress. The research of Whang and Lee shows that stress is significantly related to Internet addiction, but the causal relationship is not pointed out (Whang and Lee, 2003); the other is to prove that Internet addiction and stress have a one-way causal relationship, and stress is one of the main reasons for college students’ Internet addiction (Zhang et al., 2015). So it is very meaningful to explore the relationship between college students’ perceived stress and Internet addiction. According to the “Compensatory Internet Use (CIU)” model, the alternative model of compensatory Internet use is based on conceptual problems and methodological flaws surrounding Internet addiction research that make theoretical development difficult (Kardefelt-Winther, 2014). The psychological literature on Internet addiction is combined with the research on the motivation of Internet use. It tries to reasonably infer the frequent assumptions that people use the Internet to avoid real-life problems or relieve irritability. This assumption sometimes leads to negative results. The theoretical argument is that by understanding how motivation regulates the relationship between psychosocial well-being and Internet addiction, we can draw conclusions about how online activities compensate for psychosocial problems. This can help explain why some people still spend a lot of time online despite experiencing negative results (Cristóbal et al., 2019).

The motivation to use the internet as a coping strategy is an important factor in the development and maintenance of internet addiction. As mentioned in Griffiths (2005), feelings of numbness or avoidance can reduce the stress of participating in activities. Internet addiction can be used as a coping mechanism to deal with negative life situations or to relieve anxiety (a negative emotional state consisting of restlessness, often accompanied by sadness, irritability and anxiety; Capponi, 2013). Therefore, based on this model, we can make a reasonable guess that there is a certain relationship between perceived stress and Internet addiction.

### Moderating Effect of Flow

Flow is a state closely related to inner psychological motivation, and is the overall feeling that an individual feels when he is fully engaged in action. People in this state are usually reluctant to be disturbed, able to forget time and fatigue, and accompanied by a high degree of excitement and fullness (Csikszentmihalyi, 1975; Wilder, 1989). When an individual feels that his skills match the challenge presented by the task at hand, a state of “flow” occurs. If the balance between skills and challenges is broken, people will feel bored or anxious (Ellis et al., 1994). There is a variety of activities that make flow happen (Chen et al., 1999). Studies have shown that many online activities will induce the flow experience, so it is appropriate to use flow theory to study Internet addiction in this study. It should be pointed out that flow experience can bring positive and negative results (Woszczynski et al., 2002). Previous studies have confirmed that flow can be associated with Internet addiction (Thatcher et al., 2007; Kim and Davis, 2009; Stavropoulos et al., 2013, 2018; Yang et al., 2014). Experiencing flow requires the premise of clear goals. Relieving negative emotions under pressure through the Internet is achieved through a pleasant internal state of concentration, which means that this can be achieved through unimpeded use of the Internet. It is possible to experience flow in an online environment without clear goals, because the Internet can be considered an inherently enjoyable environment (Hoffman and Novak, 1996; Novak et al., 2000). Undergraduates’ psychological pressure, heart flow and coping style are significantly correlated, among which psychological pressure coping style is significantly negatively correlated; heart flow and coping style are significantly positively correlated; psychological pressure and heart flow are significantly negatively correlated (Qi, 2020). Prior to this, related studies have shown that network flow experience is an important factor in predicting Internet addiction (Yu and Zhang, 2005). And based on this, it can be speculated that if a person is more likely to experience flow online, he is more likely to use the Internet to relieve negative emotions caused by stress. Therefore, the first hypothesis of this study is that flow plays a moderating role in the effect of perceived stress on internet addiction.

### Mediation Effect of Procrastination

The relationship between the Internet and perceived stress is also related to its use as a tool for procrastination. Procrastination is defined as delaying a behavior until subjective discomfort is experienced (Ferrari et al., 1975; Solomon and Rothblum, 1984; Lay, 1986). It is a general feature or characteristic based on failure of self-regulation (Svartdal et al., 2016). Academic procrastination is a special phenomenon of lack of academic performance, which is common among undergraduates all over the world (Rabin et al., 2011). The Internet provides many pastimes and is always described as a means of entertainment. Through it, individuals can obtain a pleasant, interesting and entertaining experience, which helps to relieve perceptual stress (Lavoie and Pychyl, 2001). Naturally, the Internet is considered to be a good tool for relieving perceived stress and a promoter of procrastination (Davis et al., 2002), which means that people will use the Internet to deliberately delay their actions, although this delay may lead to negative consequences. CIU correlated significantly with mean hours online (Durkee et al., 2012). When a person often uses the Internet for procrastination, it is more likely to suffer the negative consequences of using the Internet, one possibility is Internet addiction, correlation analysis revealed that Internet addiction was positively correlated with procrastination (Geng et al., 2018). In addition, there are two possible behavioral tendencies when individuals are in a stressful and bad emotional state. One is to actively seek solutions to remove pressure; the other is to use procrastination to temporarily escape pressure, because procrastination is a common behavioral trait based on self-regulatory failure (Dietz et al., 2007; Steel, 2007; Steel and Klingsieck, 2016), and the network is a tool for carrying out procrastination. Therefore, based on the relationship between perceived stress and Internet addiction, the procrastination variable is added to this study. Thus, the second hypothesis of this study is that perceived stress affects Internet addiction through the mediating effect of procrastination.

The purpose of this study is to explore whether there is a relationship between perceived stress and Internet addiction, and hypothesize that procrastination plays a mediating role and flow plays a moderating role in this relationship.

## Materials and Methods

### Participants

In this study, data were collected from 460 college students. All participants gave informed consent. The questionnaires lacking basic information and incorrectly filled out were eliminated, and finally 446 valid questionnaires were obtained, including 163 male students (36.5%) and 283 female students (63.5%). The sample of college students ranged from grade 1 to grade 4. Freshmen accounted for 25.3%, sophomores accounted for 25.8%, juniors accounted for 31.6%, and seniors accounted for 17.3%.

### Measures

#### Chinese Perceived Stress Scale (CPSS)

PSS is a self-assessment scale for measuring perceived stress. So far, the scale includes three versions of PSS-14, PSS-10, and PSS-4. As scholars from various countries pay more attention to direct stress and individual mental health, PSS was introduced into more than 20 countries including France, Germany, and China after cross-cultural debugging. Professor Yang Tingzhong et al. first Chineseized PSS-14 and introduced it to China in 2003 (Yang and Huang, 2003). The Cronbach’s a of the Chinese version of Perceptual Stress Scale (Chinese PSS, CPSS) is 0.78, and the correlation coefficient between each item and the total score is 0.37∼0.53, which means the scale has good credibility. This scale consists of 14 items, which is used to measure subjects’ subjective stress perception levels. There are six forward questions and 8 reverse questions. The reverse scoring questions are 4, 5, 6, 7, 9, 10, 12, and 13. The scale has two dimensions, namely tension and sense of loss of control. A 5-point scoring method is used. Finally, the total score of the statistical scale is calculated. The higher the score, the more obvious the psychological pressure of the subjects. The method of calculating the score is: “Never” scores 1 point, “Occasionally” scores 2 points, “Sometimes” scores 3 points, “Always” scores 4 points, and “Always” scores 5 points. Compared with other stress scales, this scale has fewer items and easier response, which is applicable to the evaluation of college students’ mental health. Likert grade 5 score was used in the scale, CPSS in the study sample had good reliability and validity.

#### Internet Addiction Scale

The Internet Addiction Test (IAT) was developed by Young, a scholar of the University of Pittsburgh, in 1995, based on the DSM-IV standard and based on the diagnosis of pathological gambling addiction. Whether Internet addiction and the degree of Internet addiction. The scale consists of 20 items and uses a 5-point scoring method. These items involve compulsive Internet surfing and Internet addiction withdrawal, Internet addiction tolerance, interpersonal health, time management, etc. The total score is 100 points, the higher the score, the more serious the degree of Internet addiction. Kronbach’s Alpha is 0.904, which is a broad measurement tool for Internet addiction. It has been translated into multiple languages and its psychometric properties have been tested in different samples around the world. Therefore, it has good reliability.

#### Procrastination

In order to directly measure the frequency of procrastination in using the Internet, a project was used (do you often use the Internet to postpone tasks that you feel unpleasant?). Likert scale is used to divide the project into 1 (ever) to 5 (often). This project focused on the core aspect of procrastination, that is, voluntarily choosing the delayed task to compete with another task (Steel, 2007), which includes the emotional attitude toward the delayed task (Pychyl et al., 2000). The higher the score, the easier it is for the participant to procrastinate through the use of the Internet. There was no missing value in this project.

#### Flow Scale

A scale in the (Novak et al., 2000) network flow model is used in this study. The scale has 12 items, which are divided into three dimensions, namely, concentration, sense of presence, and time distortion. Using a Likert 7 grade score, Cronbach’s Alpha of 0.755, which has good reliability.

### Statistical Tools

SPSS 24.0 is used for descriptive statistics and correlation analysis, and the process program prepared by Hayes is used to test the mediating and moderating effect.

## Results

### Common Methodology Deviation

In this study, the questionnaire survey method is used to collect data. In the process of data statistics, Harman single factor test is used to estimate the influence of common method deviation. Exploratory factor analysis is carried out on all the questions of the four variables. Eight factors with characteristic roots greater than 1 are extracted from the results, and the maximum factor variance interpretation rate is 25.31%, which is less than the 40% limit proposed by Podsakoff et al., so there is no serious common method deviation in this study.

### Descriptive Statistics and Correlation Analysis

As is shown in the Table 1, perceptual stress is significantly positively correlated with Internet Addiction, flow and procrastination; Internet addiction is significantly positively correlated with flow and procrastination; there is a significant positive correlation between flow and procrastination.

**TABLE 1:** The Results of descriptive statistics and correlation analysis.

	*M*	*SD*	Perceived stress	IA	Flow	Procrastination
Perceived stress	2.8681	0.43578				
IA	2.3785	0.70133	0.444**	1		
Flow	4.1171	0.96192	0.305**	0.591**	1	
Procrastination	2.935	1.09042	0.359**	0.553**	0.448**	1

**p < 0.01; IA, Internet addiction.

### Mediation Model

First, Model 4 in Process (Model 4 is a simple mediation model) compiled by Hayes (2012) is used to test the mediation effect of procrastination between perceived stress and Internet addiction under the control of gender and grade. The results (Table 2) showed that the predictive effect of perceived stress on Internet addiction was significant (*t* = 10.49, *p* < 0.01), and the direct predictive effect of perceived stress on Internet addiction was still significant (*t* = 7.12, *p* < 0.01) after the mediating variables were added. Perceived stress has a significant positive predictive effect on procrastination (*t* = 7.97, *p* < 0.01), and procrastination has a significant positive predictive effect on Internet addiction (*t* = 11.33, *p* < 0.01). In addition, the upper and lower limits of the 95% confidence interval of perceived stress on Internet addiction and procrastination mediation effect do not contain 0 (Table 3), which indicates that perceived stress cannot only directly predict Internet addiction, but also predict procrastination behavior through procrastination mediation effect. The direct effect (0.462) and the mediating effect (0.261) accounted for 63.94 and 36.06% of the total effect (0.723) respectively.

**TABLE 2:** Mediation model test of procrastination.

Regression equation		Fitting indicators		Significance	
			
Outcome variable	Predictive variable	*R*^2^	*F* (*df*)	*t*	*p*
IA		0.45	37		
	Gender			−1.36	0.17
	Grade			0.02	0.98
	Perceived stress			10.49	0.00**
Procrastination		0.36	22.19		
	Gender			0.91	0.36
	Grade			−0.14	0.89
	Perceived stress			7.97	0.00**
IA		0.62	67.81		
	Gender			−2.03	0.04
	Grade			0.10	0.92
	Procrastination			11.33	0.00**
	Perceived stress			7.12	0.00**

**p < 0.01; IA, Internet addiction.

**TABLE 3:** Total effect, direct effect, and mediating effect.

	Effect	BootSE	BootLLCI	BootULCI	Percentage
Total effect	0.723	0.076	0.573	0.873	
Direct effect	0.462	0.071	0.322	0.598	63.94
Mediating effect	0.261	0.047	0.172	0.355	36.06

### Moderation Model

The model1 in process is used to test the moderating effect of flow between perceived stress and Internet addiction. Gender and grade are included in the control variable. As shown in Table 4, the positive predictive effect of perceived stress on Internet addiction is significant (*t* = 7.695, *p* < 0.01), and it is still significant when flow variables are included (*t* = 7.173, *p* < 0.01). Flow has a significant positive predictive effect on Internet addiction (*t* = 13.100, *p* < 0.01), and the interaction term of perceived stress and flow is of great importance in predicting Internet addiction (*t* = 2.243, *p* < 0.01). This suggests that flow has a significant moderating effect on the relationship between perceived Stress and Internet addiction.

**TABLE 4:** Regulating model test of flow.

	IA		IA	
		
	*t*	*p*	*t*	*p*
Perceived stress	7.695	0.00**	7.173	0.00**
Flow	13.284	0.00**	13.100	0.00**
Interaction			2.243	0.025**
*R*^2^	0.426		0.432	
*F*	164.323		112.223	

**p < 0.01; IA, Internet addiction.

**FIGURE 1:**
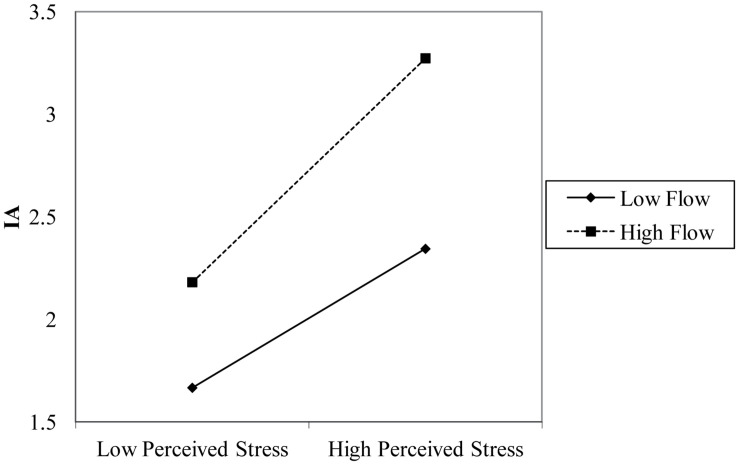
A simple slope graph of perceived stress and Internet addiction.

A simple slope test was performed to explain more clearly the interaction between the perceived stress and the flow. The effect analysis diagram found that perceived stress has a significant positive predictive effect on Internet addiction, regardless of low and high flow. While, the positive predictive effect of high flow is more obvious (Figure 1).

## Discussion

This study links perceived stress and procrastination with internet addiction, which has certain theoretical significance. Exploring the mediating role of procrastination between perceived stress and Internet addiction is helpful to study the reasons that affect college students’ Internet addiction, and it also provides a new analysis perspective for college students’ procrastination behavior. Procrastination is a deliberate and unnecessary act of delay, which can be manifested in trivial matters of daily life, but serious or frequent procrastination often leads to serious consequences. College students are prone to procrastination and Internet addiction. When people feel stressed, they may develop bad emotions and take negative responses, which tends to be procrastination. College students, as a large group exposed to the Internet, can easily use the Internet as a tool for procrastination. Therefore, this negative coping style of negative emotions will lead to the consequences of negative behavior, that is, Internet addiction. The problems that college students may have in the process of using the Internet have aroused widespread concern. This study points out that perceived stress urges individuals to use procrastination to escape bad emotions, which in turn leads to college students’ Internet addiction.

Based on the emotional regulation theory and the structural view of emotional regulation, Kim’s experimental results show that individuals have the ability to perform certain emotional behaviors in an appropriate way and avoid the ability to perform certain emotional behaviors in an inappropriate way (Kim and Davis, 2009). This study constructs a moderating model to explore the moderating role of flow on the network in the relationship between perceived stress and Internet addiction. The results show that the flow on the network does play a moderating role, which also validates the first hypothesis of this study. Perceived stress leads to negative emotions, the generation of negative emotions prompts individuals to seek positive stimuli. As an inherent pleasant environment, the Internet will bring pleasure to the flow experience when using the Internet, and make people become an internet addict due to long time indulging in internet fun. Specifically, it is found that individuals who are more easier to produce flow experience on the Internet are more likely to produce Internet addiction than those who are less likely to produce flow experience, which indicates that there are individual differences in the mechanism of forming Internet addiction. In addition, this study also provides a new perspective for the flow experience, that is, flow experience may have a negative impact, while the flow experience in the network environment brings good experience to individuals, and there are some potential problems such as addictive behavior.

In addition, this study has certain practical significance. The mediating and moderating model constructed in this study have certain enlightenment to guide college students to make rational use of the Internet and reduce the occurrence of Internet addiction caused by individual stress. First of all, college students should be encouraged to timely detect stress levels, reduce negative responses to negative emotions, and actively seek help in the face of bad emotions; secondly, college students should use the Internet moderately and regard the Internet as a tool to assist social contact and study, instead of a tool to procrastinate, so as to reduce the negative impact of Internet addiction; finally, cognitive intervention can be carried out to reduce the time and frequency of network use of individuals who are easy to have flow experience on the Internet.

## Limitations

There are also some shortcomings in this study, which need to be improved in the future research. First of all, in order to avoid the influence of other irrelevant factors, a project may be needed to more directly and clearly measure the level of flow in network usage. This study uses a cross-sectional design to investigate the relationship between procrastination and Internet addiction, but this may affect the direction of measurement. There may be a two-way effect between procrastination and Internet addiction, that is, Internet addiction will also aggravate procrastination, which needs to be explored by longitudinal studies and hierarchical linear modeling in the future.

## Conclusion

Based on the compensatory network use model and emotion regulation theory, this study constructs two models with procrastination as the mediating variable and flow on the Internet as the moderating variable, which clarifies how procrastination affects college students’ procrastination behavior (the mediating effect of procrastination), and explains under which conditions perceived stress has a more significant effect on Internet addiction (the moderating effect of heart flow). The results are consistent with the hypothesis. The perceived stress of college students is significantly related to Internet addiction, in which procrastination plays a mediating role in it, and the flow on the Internet plays a moderating role. The results of this study combined with the actual situation of college life, link perceived stress with procrastination and flow, and explore reasons behind Internet addiction among college students. This research has positive significance for expanding the study of the relationship between perceived stress and Internet addiction and constructing a theoretical model of Internet addiction.

## Data Availability Statement

The raw data supporting the conclusions of this article will be made available by the authors, without undue reservation.

## Ethics Statement

The studies involving human participants were reviewed and approved by the Institutional Review Board, Normal College, Qingdao University. The participants provided their written informed consent to participate in this study. The patients/participants provided their written informed consent to participate in this study.

## Author Contributions

ZG: propose research ideas and design research schemes. LW: responsible for conducting experiments. ZG and LW: responsible for collecting, cleaning, and analyzing data and responsible for manuscript drafting. HW: responsible for final revision. All authors contributed to the article and approved the submitted version.

## Conflict of Interest

The authors declare that the research was conducted in the absence of any commercial or financial relationships that could be construed as a potential conflict of interest.
